# A Global Perspective on Trends in Nature-Based Tourism

**DOI:** 10.1371/journal.pbio.1000144

**Published:** 2009-06-30

**Authors:** Andrew Balmford, James Beresford, Jonathan Green, Robin Naidoo, Matt Walpole, Andrea Manica

**Affiliations:** 1 Department of Zoology, University of Cambridge, Cambridge, United Kingdom; 2 Conservation Science Program, World Wildlife Fund–US, Washington, DC, United States of America; 3 Fauna and Flora International, Cambridge, United Kingdom; The David and Lucile Packard Foundation, United States of America

## Abstract

Reports of rapid growth in nature-based tourism and recreation add significant weight to the economic case for biodiversity conservation but seem to contradict widely voiced concerns that people are becoming increasingly isolated from nature. This apparent paradox has been highlighted by a recent study showing that on a per capita basis, visits to natural areas in the United States and Japan have declined over the last two decades. These results have been cited as evidence of “a fundamental and pervasive shift away from nature-based recreation”—but how widespread is this phenomenon? We address this question by looking at temporal trends in visitor numbers at 280 protected areas (PAs) from 20 countries. This more geographically representative dataset shows that while PA visitation (whether measured as total or per capita visit numbers) is indeed declining in the United States and Japan, it is generally increasing elsewhere. Total visit numbers are growing in 15 of the 20 countries for which we could get data, with the median national rate of change unrelated to the national rate of population growth but negatively associated with wealth. Reasons for this reversal of growth in the richest countries are difficult to pin down with existing data, but the pattern is mirrored by trends in international tourist arrivals as a whole and so may not necessarily be caused by disaffection with nature. Irrespective of the explanation, it is clear that despite important downturns in some countries, nature-related tourism is far from declining everywhere, and may still have considerable potential both to generate funds for conservation and to shape people's attitudes to the environment.

## Abstract 2

Falling attendance at United States and Japanese national parks has led to claims of a pervasive shift away from nature-based recreation. A global analysis, however, now suggests that while visit rates are declining slightly in some richer countries, elsewhere nature tourism is booming.

## Author Summary

Nature-based tourism is frequently described as one of the fastest growing sectors of the world's largest industry, and a very important justification for conservation. However, a recent, high profile report has interpreted declining visit rates to US and Japanese national parks as evidence of a pervasive shift away from nature tourism. Here we use the largest database so far compiled on trends in visits to Protected Areas around the world to resolve this apparent paradox. We find that, while visit rates—measured in two different ways—are indeed declining in some wealthy countries, in roughly three-quarters of the nations where data are available, visits to Protected Areas are increasing. Internationally, rates of growth in the number of visits to such areas show a clear negative association with per capita income, which interestingly is matched by trends in foreign arrivals as a whole. Our results therefore suggest that, despite worrying local downturns, nature-related tourism is far from declining everywhere, and may still have considerable potential to generate funds for conservation and engage people with the environment.

## Introduction

Across southern Africa, nature-based tourism reportedly now generates roughly the same revenue as farming, forestry, and fisheries combined [1]. Worldwide, tourism as a whole has been estimated to account for roughly 10% of gross domestic product (GDP) [2], with wildlife viewing and outdoor recreation (much of it centred on protected areas [PAs]) reportedly making up one of its fastest growing sectors [3]–[5]. Though statistics like these are rarely supported by detailed data, they underpin widespread recognition that nature-based tourism is an important ecosystem service [6], capable of generating substantial resources for both conservation and local economic development [3],[7],[8]. This is particularly significant given that PAs are under increasing pressure to provide economic justification for their existence [9]–[12].

This positive perspective stands in sharp contrast to growing concerns about an emerging disconnect between people and their natural environments. Increasing urbanisation and the rise of sedentary, indoor pastimes (such as television, the Internet, and video games) have been linked to a reduction in informal, outdoor recreation (Pyle's “Extinction of Experience” [13]), with potentially serious consequences for childhood development, mental and physical wellbeing, and environmental knowledge and concern [14]–[21]. Many see this as a major challenge for biodiversity conservation [13],[14],[21],[22]: if people no longer experience and know their natural environments, how can they be expected to care about them?

These worries have been further fuelled by a recent and widely publicised paper examining trends in 16 measures of outdoor recreation (14 from the United States, plus one each from Japan and Spain [23]). This analysis showed that, expressed per head of population, visits to natural areas in the United States and Japan (as well as participation in duck-hunting and fishing in the United States, but not hiking, camping, or other hunting) have declined since the late 1980s (though for contrasting US figures, see [24]). From these per capita trends the authors conclude there has been “…a fundamental and pervasive shift away from nature-based recreation” [23; see also 21,25]. However, the paper produced no evidence of declines outside the United States and Japan (and per capita national park attendance in Spain, the only other country sampled, has not declined), raising the possibility that the reported shift may not be universal.

To date, lack of data has meant no study has looked at trends in nature-based tourism across more than a handful of countries. Here, we use newly compiled information on visitor numbers to 280 PAs in 20 countries (Australia, Canada, Chile, China, Ecuador, Ghana, India, Indonesia, Japan, Korea, Madagascar, Peru, Philippines, Rwanda, South Africa, Sri Lanka, Tanzania, Uganda, UK, United States) between 1992 and 2006 to explore the generality of the United States and Japan results and to understand the apparent mismatch with the claim that globally, nature-based tourism is on the rise. Importantly, because we are interested in trends in nature tourism as a whole as well as individual interest in nature, we analyse changes in both total visit numbers and visit numbers corrected for national population size. The latter are a better reflection of per capita interest in a country's PAs [23], but the former are a more sensible proxy for trends in the overall benefit derived from nature tourism as an ecosystem service.

## Results

Our analysis of standardised rates of change in PA visit numbers provides limited support for the previously reported declines in nature-based activities in the United States. Using total visit numbers, only 14 out of 51 US PAs for which we could get data showed significant decreases in visit number (at *p*<0.05), while 11 exhibited significant increases. Adjusting for changes in national population size, the number of US PAs experiencing significant declines rose to 27 and the number with increasing attendance fell to just 6. Clearly, the decline in per capita visitation to US PAs we could sample is real, but arises largely because absolute attendance has been almost static despite a growing national population. In Japan, the only PA for which we had data showed a nonsignificant decline in visits, whether expressed in terms of total or per capita visit numbers.

**Figure 1:**
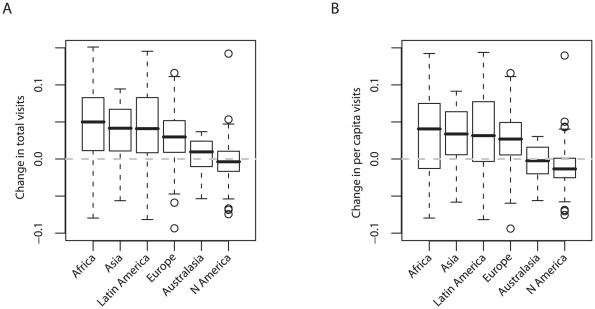
Comparisons across continents of rates of change in numbers of visits to protected areas, 1992–2006; lines, boxes, error bars, and circles show medians, interquartile ranges, minima and maxima (excluding outliers), and outliers (which deviate from the median by >1.5× interquartile range), respectively. (A) Changes in total visit number; (B) changes in per capita visit number.

More interestingly, these weak declines in two countries are far from globally typical: instead, visitor trends show marked geographical variation. When we pooled standardised rates of change within continents, rather than being negative we found that trends in total visit numbers were not significantly different from zero in North America or Australasia, and were on average positive in Africa, Europe, Asia, and Latin America (Figure 1A; *F*_5,274_ = 10.2, *p*<0.001; in post hoc tests only Australasia and North America had rates of changes not significantly different from zero at *p*<0.05). There was similar broad-scale variation when we compared trends in per capita visit numbers across continents (Figure 1B: *F*_5,274_ = 10.4, *p*<0.001, with significant positive trends again everywhere apart from Australasia and North America).

These patterns of spatial heterogeneity were confirmed when data were analysed by country (Table S1). Total visit numbers to PAs on average grew in 15 out of the 20 countries sampled and fell in four (with Uganda showing no change). Even allowing for population growth, per capita visit numbers rose in 14 countries (with Uganda and Australia added to the list of countries showing falling visitation). The only country we sampled outside the Organisation for Economic and Co-Operation Development (OECD) with consistently falling PA visitation was Indonesia.

**Figure 2:**
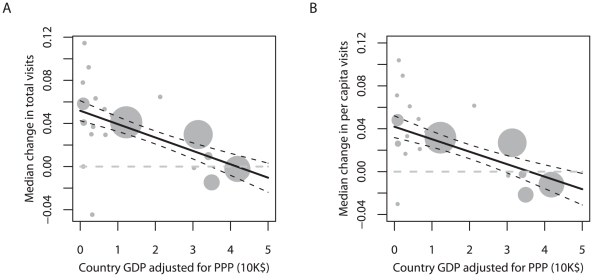
Median national rates of change in numbers of visits to PAs in relation to per capita GDP (in 2005), adjusted for PPP; the number of PAs sampled per country is reflected in point size, and used to weight the regression; solid line represents the best model, dark dashed lines represent ±1 standard error (SE). (A) Changes in total visit number; (B) changes in per capita visit number.

National rates of change are closely associated with wealth. In contrast to the United States and Japan, poorer countries typically had increasing numbers of PA visits, with median standardised rates of growth in total visit numbers showing a clear negative relationship with per capita GDP (Figure 2A; regression weighted by number of PAs sampled per country: adjusted *r* ^2^ = 0.52, *n* = 20 countries, *F*_1,18_ = 21.8, *p*<0.001). This result was not due to correlated variation in population growth, because the negative link with rising wealth held when visit numbers were adjusted for changes in population size (Figure 2B; weighted regression of median standardised rates of change in per capita visit numbers against per capita GDP: adjusted *r* ^2^ = 0.43, *n* = 20, *F*_1,18_ = 15.5, *P*<0.001). As a further check for any confounding effects of population growth, we compared changes in total visit numbers with national population growth rates, but found no association between the two (Figure S1; weighted regression: adjusted *r* ^2^ = 0.07, *n* = 20, *F*_1,18_ = 2.6, NS). The tendency for PA visitation to be increasing in poorer countries appears to be independent of population growth.

## Discussion

Our dataset on PA visits has far broader geographical coverage than any others we are aware of, yet yielded no evidence to support the idea of a consistent global decline in nature-based recreation. Instead it appears that falling visitation is mostly restricted to a few well-off countries. When we adjusted visit numbers for population growth to examine individual participation in nature recreation we were able to replicate previously reported declines in per capita visit number in the United States and Japan [23], but also found that in most other countries population-adjusted visit numbers have been increasing.

These patterns were more marked when we looked at trends in PA visitation as a whole, using total numbers of PA visits. We found these are growing on four out of six continents and in 15 of the 20 countries for which we could get data. These changes in average visit rates are quite well predicted by wealth, but are unrelated to national population growth—confirming the finding from the per capita analysis that it is not the case that visitation is increasing simply where populations are growing rapidly. Instead, it appears that PA visitation is generally growing, but at a progressively lower rate (eventually falling below zero) with rising affluence.

We do not have a ready explanation for this negative link between visit growth and wealth, and believe this will be hard to unravel from correlational analyses alone. It could be related to the emergence of “videophilia” [20], or to other aspects of growing urbanisation or increasingly sedentary lifestyles [14]–[19],[21]. These ideas are plausible, but direct evidence for them is sparse. Given that very many potential drivers co-vary with one another and with time, causality may be difficult to establish until more detailed data become available, or an experimental approach is adopted.

One nonexclusive alternative explanation for the patterns of changing PA visitation that we see could be that many formal protected areas in richer countries are becoming increasingly crowded and thus less attractive to nature enthusiasts (J. du Toit, personal correspondence). Overcrowding and the perception of overcrowding have been noted as a concern of visitors to many larger US National Parks for over a decade [26],[27]. If would-be visitors are instead switching to less publicised sites where visitors are not counted, overall visit rates to natural areas in these countries could be stable or even growing, yet still recorded as declining.

**Figure 3:**
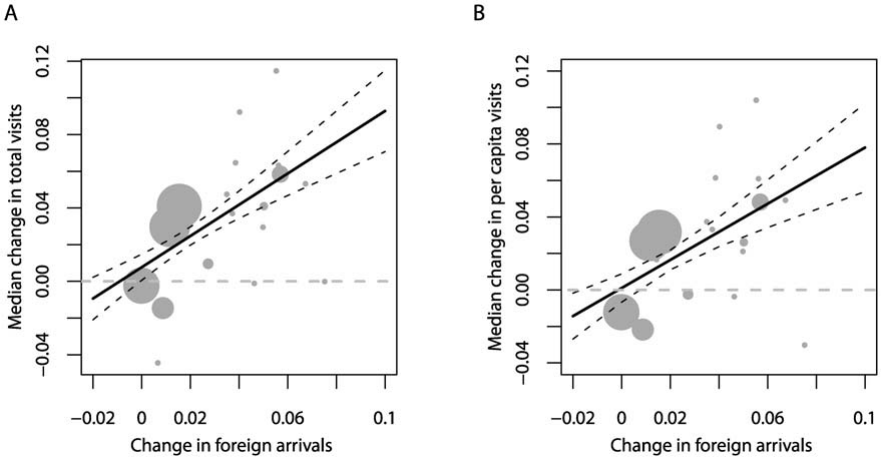
Median national rates of change in numbers of visits to PAs in relation to standardised annual change in foreign arrivals (1995–2005); symbols as in Figure 2. (A) Changes in total visit number; (B) changes in per capita visit number.

One other explanation for the pattern we see could be that there is a shift in preference away from domestic destinations as nature-focused tourists become wealthier and alternative wildlife attractions in less costly developing countries become more accessible [28],[29]. Strikingly, the patterns we uncovered for PA visitor trends are echoed by those for international tourism more generally: standardised national rates of change in all foreign arrivals (from [30]) co-vary positively with median changes in total and per capita PA visit numbers (for total visit numbers, Figure 3A; regression weighted as in Fig. 2A, *r^2^* = 0.34, *n* = 19 countries excluding Rwanda, for which no arrival information was available: *F*_1,17_ = 10.2, *p*<0.01; for per capita visit numbers, Figure 3B; weighted regression: *r^2^* = 0.25, *n* = 19, *F*_1,17_ = 7.0, *p*<0.05). Changes in foreign arrivals also show a negative relationship with per capita GDP (Figure S2; *r^2^* = 0.29, *n* = 19, *F*_1,17_ = 8.2, *p* = 0.01), falling to zero growth in the United States. These results suggest that trends in nature-based recreation might be less driven by attitudes to nature per se and more to do with how rising wealth and the emergence of new destinations influence the dynamics of recreation as a whole [31],[32]. To resolve this, more data would be needed than we were able to obtain on the nationalities and motivations of visitors to individual PAs.

Regardless of the underlying drivers, our analyses indicate that it is premature to conclude that PA visit data indicate a general and pervasive shift away from nature tourism. This is apparently occurring in a few developed countries, where it is worrying, and where it certainly demands more attention. But in contrast, in most developing countries visits to protected areas are growing at rates that mirror general increases in tourism and travel—in many cases by more than 4%/y (Figure 2A). This is especially significant for conservation, given that, unlike other nonconsumptive uses of ecosystems, nature-based tourism produces tangible financial flows that can, if carefully developed, be of direct benefit to local decision-makers [7]–[9],[33],[34].

Tourism can often provide a strong incentive for protection in biodiversity-rich areas [8], and formal designation of such sites can raise their profile and influence tourism visitation [35]. However, increasing visitor numbers alone is no guarantee that tourism revenues will be reinvested in conservation [36]. Equally, recording visitor numbers does not equate with the much less common practices of monitoring or managing tourism impacts [37]. International nature tourism raises other important worries—about CO_2_ emissions, about its vulnerability to changing fashions, about disturbance to wildlife and nearby people, and about how far its revenues filter down to local communities [24],[34],[38]–[41]. Nature-based tourism is only likely to be sustainable under certain conditions of effective planning, management, and local participation [7],[42]–[44]. However, to the extent that these concerns can be addressed, our results argue that far from having a diminishing role, nature-based recreation has the potential in many parts of the world to make a growing contribution to both conservation and sustainable development.

## Materials and Methods

Somewhat surprisingly, there is no global database or consistent set of national statistics summarising trends in nature-based tourism. Instead, like previous authors [23] we infer changes in the sector as a whole from visits to PAs. We compiled information on annual visitor numbers to terrestrial PAs (including any listed in [45]). PA visits are among the most frequent forms of nature-based recreation recorded in the United States [23], and we suggest they are likely to account for an even greater proportion of nature recreation in other countries, where alternatives are less developed. We collected data from as many sources as possible: the grey and published literature, personal contacts, and especially the World Wide Web. The methods used to record visitors were rarely reported in detail, but varied widely, including dedicated studies, gate receipts, and traffic counts [46]. There are also likely to be biases in some datasets, with corruption, for example, perhaps leading to systematic under- (and in some cases, maybe over-) reporting of visitor numbers [47]. These problems may confound estimation of absolute visitor numbers, but will have less impact on within-PA changes in visitor numbers over time, and so here we used all available information.

In total we were able to collate ≥6 y of data (between 1992 and 2006) for 280 PAs from 20 countries. We then expressed visitation trends at each PA in two ways—using total visit number, as a measure of the overall tourism benefit provided by the PA; and (as in [23]) using visit number divided by national population size in that year (from [30]), as a measure of per capita use of the PA. For PAs with large numbers of nondomestic visitors, tracking per capita use by dividing by national population size is imperfect (and data on visitor origins are too patchy for any more sophisticated adjustment by population size). However, data we obtained for 190 PAs (many lacking time series information and so excluded from our core analysis) indicate that, for all except one continent, a mean of >70% of visitors are nationals, so that errors caused through adjusting by national population size are relatively limited. The exception is Africa, where on average only ∼30% visitors are nationals. For this continent, adjustment by national population growth (which is also generally higher than elsewhere) is probably excessive and so negatively biases estimates of trends in per capita visit rates.

For each PA we next performed linear regressions of total visit number and per capita visit number on year, and derived standardised measures of rates of change (ranging from +1 to −1) as (slope/maximum total [or per capita] visit number predicted by the regression during the 15-y range). We explored geographical variation in trends in our two measures of visit numbers by calculating median standardised rates of change across continents, and across countries (Table S1). We compared the latter with per capita GDP adjusted for purchasing power parity (PPP) (for 2005, from [30]), using linear regression weighted by the number of PAs sampled in each country. As an additional check to see whether our results for total visit number were confounded by changes in national population size, we performed an equivalent weighted regression of national median change in total visit number versus annual population growth (for 1990–2006, from [30]). Last, to see whether our findings were specific to nature-related tourism, we also obtained data on trends in all foreign arrivals between 1995 and 2005 (again from [30]), and compared standardised national rates of change (calculated in the same way as for PA visits) with per capita GDP and with median standardised rates of change in total visit numbers.

## Supporting Information

Figure S1 **Median national rates of change in total numbers of PA visits in relation to annual population growth (1990–2006); the number of PAs sampled per country is reflected in point size.**
(0.53 MB EPS)

Figure S2 **Standardised annual change in foreign arrivals (1995–2005) in relation to per capita GDP (in 2005), adjusted for PPP; solid line represents the best model, dashed lines represent ±1 standard error (SE).**
(0.58 MB EPS)

Table S1 **National values of annual rates of change in total and per capita visits to PAs, per capita GDP, number of PAs sampled, and annual rates of change in foreign arrivals.**
(0.05 MB DOC)

## Abbreviations

GDP: gross domestic product
PA: protected area
PPP: purchasing power parity

## References

[1] Scholes RJ, Biggs R (2004). Ecosystem services in southern Africa: a regional perspective.

[2] World Travel and Tourism Council (2007). The global travel and tourism summit.

[3] Goodwin HJ (1996). In pursuit of ecotourism.. Biodiv Conserv.

[4] Mastny L (2001). Treading lightly: new paths for international tourism.

[5] Davenport L, Brockelman WY, Wright PC, Ruf K, Rubio del Valle FB, Terborgh J, van Schaik C, Davenport L, Rao M (2002). Ecotourism tools for parks.. Making parks work.

[6] Millennium Ecosystem Assessment (2005). Ecosystems and human wellbeing: biodiversity synthesis.

[7] Boo E (1990). Ecotourism: the potentials and pitfalls.

[8] Gossling S (1999). Ecotourism: a means to safeguard biodiversity and ecosystem functions?. Ecol Econ.

[9] Ceballos-Lascurain H (1996). Tourism, ecotourism and protected areas.

[10] Walpole MJ, Goodwin HJ, Ward KGR (2001). Pricing policy for tourism in protected areas: lessons from Komodo National Park, Indonesia.. Conserv Biol.

[11] Wilkie DS, Carpenter JF (2002). Can nature tourism help finance protected areas in the Congo Basin?. Oryx.

[12] West P, Igoe J, Brockington D (2006). Parks and peoples: the social impact of protected areas.. Ann Rev Anthropol.

[13] Pyle RM (2003). Nature matrix: reconnecting people and nature.. Oryx.

[14] Pyle R (1993). Thunder tree: lessons from a secondhand landscape.

[15] Nabhan GP, Trimble S (1994). The geography of childhood: why children need wild places.

[16] Balmford A, Clegg L, Coulson T, Taylor J (2002). Why conservationists should heed Pokémon.. Science.

[17] Kahn PH, Kellert SR (2002). Children and nature.

[18] Barnes S (2007). How to be wild.

[19] Louv R (2005). Last child in the woods.

[20] Zaradic PA, Pergams ORW (2007). Videophilia: implications for childhood development and conservation.. J Dev Proc.

[21] Kareiva P (2008). Ominous trends in nature recreation.. Proc Natl Acad Sci U S A.

[22] Balmford A, Cowling R (2006). Fusion or failure? The future of conservation biology.. Conserv Biol.

[23] Pergams ORW, Zaradic PA (2008). Evidence for a fundamental and pervasive shift away from nature-based recreation.. Proc Natl Acad Sci U S A.

[24] Reed SE, Merenlender AM (2008). Quiet, nonconsumptive recreation reduces protected area effectiveness.. Conserv Lett.

[25] Williams N (2008). Backs to nature.. Curr Biol.

[26] Anonymous (2007). Rating the parks.. Consumer Reports.

[27] Fretwell HL, Podolsky MJ (2003). A strategy for restoring America's National Parks.. Duke Environmental Law and Policy Forum.

[28] Ceballos-Lascurain H, Lindberg K, Hawkins DE (1993). Ecotourism: a guide for planners and managers.

[29] Prosser R, Cater E, Lowman G (1994). Societal change and the growth in alternative tourism.. Ecotourism, a sustainable option?.

[30] World Bank (2008). World development indicators 2008.

[31] Butler R (1980). The concept of a tourism area cycle of evolution.. Can Geogr.

[32] World Tourism Organization/Organisation Mondiale du Tourisme (2002). Tourism and poverty alleviation.

[33] Walpole MJ, Leader-Williams N (2001). Masai Mara tourism reveals partnership benefits.. Nature.

[34] Walpole MJ, Thouless CR, Woodroffe R, Thirgood S, Rabinowitz A (2005). Increasing the value of wildlife through non-consumptive use? Deconstructing the myths of ecotourism and community-based tourism in the tropics.. People and wildlife: conflict or coexistence?.

[35] Buckley R (2004). Effects of World Heritage listing on tourism to Australian National Parks.. J Sust Tour.

[36] Wells MP (1993). Neglect of biological riches - the economics of nature tourism in Nepal.. Biodivers Conserv.

[37] Buckley R, Robinson J, Carmody J, King N (2008). Monitoring for management of conservation and recreation in Australian protected areas.. Biodivers Conserv.

[38] Bookbinder MP, Dinerstein E, Rijal, Cauley H, Rajouria A (1998). Ecotourism's support of biological conservation.. Conserv Biol.

[39] Butynski TM, Kalima J, Milner-Gulland EJ, Mace R (1998). Gorilla tourism: a critical look.. Conservation of biological resources.

[40] Walpole MJ, Goodwin HJ (2000). Local economic impacts of dragon tourism in Indonesia.. Ann Touris Res.

[41] Kiss A (2004). Is community-based ecotourism a good use of biodiversity conservation funds?. TREE.

[42] Kruger O (2005). The role of ecotourism in conservation: panacea or Pandora's box?. Biodivers Conserv.

[43] Eagles PFJ (2009). Governance of recreation and tourism partnerships in parks and protected areas.. J Sust Tour.

[44] Plummer R, Fennell DA (2009). Managing protected areas for sustainable tourism: prospects for adaptive co-management.. J Sust Tour.

[45] World Conservation Union and United Nations Environment Programme-World Conservation Monitoring Centre (2007). World database on protected areas, version 2007.

[46] Eagles P (2002). Trends in park tourism: economics, finance and management.. J Sust Tour.

[47] Cochrane J (2003). Ecotourism, conservation and sustainability: a case study of Bromo Tengger Semeru National Park, Indonesia. [Unpublished PhD thesis].

