# Supplementary material for: A Global Perspective on Trends in Nature-Based Tourism
Source: PLoS Biol. 2009 Jun 30;7(6):e1000144. doi: 10.1371/journal.pbio.1000144 (PMC2694281; doi:10.1371/journal.pbio.1000144)
Supplement: Table S1 — National values of annual rates of change in total and per capita visits to PAs, per capita GDP, number of PAs sampled, and annual rates of change in foreign arrivals. (0.05 MB DOC) [file pbio.1000144.s003.doc]

**Table S1.** National values of annual rates of change in total and *per capita* visits to PAs, *per capita* GDP, number of PAs sampled, and annual rates of change in foreign arrivals.

| **Country** | **Median standardised rate of change in total PA visits** | **Median standardised rate of change in *per capita* PA visits** | ***Per capita* GDP (PPP adjusted) (US$/y)** | **No. of PAs sampled** | **Median standardised rate of change in all foreign arrivals** |
| --- | --- | --- | --- | --- | --- |
| Australia | 0.0096 | -0.0023 | 34106 | 15 | 0.0273 |
| Canada | -0.0147 | -0.0217 | 34972 | 31 | 0.0087 |
| Chile | 0.0410 | 0.0316 | 12248 | 63 | 0.0155 |
| China | 0.0632 | 0.0610 | 4088 | 2 | 0.0562 |
| Ecuador | 0.0296 | 0.0210 | 6737 | 1 | 0.0498 |
| Ghana | 0.1147 | 0.1039 | 1160 | 1 | 0.0553 |
| India | 0.0922 | 0.0895 | 2222 | 3 | 0.0403 |
| Indonesia | -0.0445 | -0.0492 | 3209 | 3 | 0.0066 |
| Japan | -0.0012 | -0.0038 | 30290 | 1 | 0.0462 |
| Korea | 0.0646 | 0.0614 | 21273 | 3 | 0.0385 |
| Madagascar | 0.0584 | 0.0480 | 834 | 24 | 0.0570 |
| Peru | 0.0532 | 0.0491 | 6452 | 1 | 0.0674 |
| Philippines | 0.0300 | 0.0167 | 2956 | 1 | 0.0143 |
| Rwanda | 0.0781 | 0.0711 | 696 | 3 | unavailable |
| South Africa | 0.0473 | 0.0373 | 8478 | 1 | 0.0349 |
| Sri Lanka | 0.0370 | 0.0332 | 3420 | 4 | 0.0372 |
| Tanzania | 0.0409 | 0.0261 | 933 | 12 | 0.0502 |
| Uganda | 0.0000 | -0.0301 | 848 | 3 | 0.0753 |
| UK | 0.0298 | 0.0269 | 31371 | 57 | 0.0116 |
| USA | -0.0023 | -0.0121 | 41813 | 51 | -0.0001 |
